# Health-related quality of life among celiacs in Portugal: a comparison between general and specific questionnaires

**DOI:** 10.3389/fimmu.2024.1372369

**Published:** 2024-03-04

**Authors:** Cláudia Chaves, Renata Puppin Zandonadi, António Raposo, Eduardo Yoshio Nakano, Fernando Ramos, Priscila Farage, Edite Teixeira-Lemos

**Affiliations:** ^1^ ESSV, Centre for Studies in Education and Innovation (CI&DEI), Polytechnic University of Viseu, Viseu, Portugal; ^2^ Faculty of Health Sciences, Nutrition Department, University of Brasília, Brasília, Brazil; ^3^ CBIOS (Research Center for Biosciences and Health Technologies), Universidade Lusófona de Humanidades e Tecnologias, Lisboa, Portugal; ^4^ Department of Statistics, University of Brasília, Brasília, Brazil; ^5^ Faculty of Pharmacy, University of Coimbra, Coimbra, Portugal; ^6^ Associated Laboratory for Green Chemistry (LAQV) of the Network of Chemistry and Technology (REQUIMTE), Porto, Portugal; ^7^ Faculty of Nutrition, Federal University of Goiás, Goiânia, Brazil; ^8^ CERNAS Research Centre, Polytechnic University of Viseu, Viseu, Portugal

**Keywords:** celiac disease, gluten-free diet, Portugal, quality of life, questionnaire

## Abstract

**Objective:**

This study aimed to compare the 36-Item Short Form Survey Instrument version 2 (SF-36-v2) (generic) and Celiac Disease Questionnaire (CDQ) (specific) questionnaires used to evaluate the quality of life (QoL) in celiac Portuguese adult individuals.

**Methods:**

This cross-sectional study used non-probabilistic sampling based on Portuguese celiac patients who accessed the online survey in 2022. The online data collection used a self-reported instrument composed of three parts: (i) socioeconomic, health, and gluten-free diet (GFD) adherence questions; (ii) SF-36 v2 – Portuguese version (generic questionnaire) and (iii) Celiac Disease Questionnaire (CDQ) (specific questionnaire).

**Results:**

A total of 234 individuals who accessed the survey completed the questionnaire. Seven of the eight SF-36 domains positively correlated to the specific questionnaire CDQ. The “General Health” domain (domain 4) showed a negative correlation with the CDQ. Differences in content between the two instruments might be able to explain this finding since the CDQ explores issues regarding the specificities of celiac disease (CD) and the lifelong GFD burden. About half of the sample from this study displayed poor diet adherence, it is possible that the SF-36 could not reflect the impact of CD treatment - the complete elimination of gluten from the diet - on patients’ health. Therefore, this issue should be carefully evaluated in future research.

**Conclusion:**

Specific validated questionnaires for CD individuals, such as the CDQ, contemplate social, economic, and clinical variables that permeate the patient’s life context. Therefore, these instruments may be more suitable for evaluating QoL in this public. However, using a general questionnaire such as the SF-36 would be indicated for comparing QOL between celiac patients and the general population or even between CD and other disease individuals. In this case, we recommend assessing GFD compliance for control parallelly.

## 1 Introduction

For Celiac disease (CD) is a permanent autoimmune disorder triggered by gluten ingestion by genetically predisposed individuals, affecting approximately 1% of the worldwide population (1, 2). CD is considered a public health problem and may cause malabsorption, leading to nutritional deficiencies, liver and bone diseases, gastrointestinal symptoms, growth deficiency, or several other consequences (1, 3, 4).

Until now, the only safe treatment for CD is a life-long gluten-free diet (GFD) (1, 3) and usually, GFD compliance improves the quality of life (QoL) in most of CD patients due to symptom remission, nutritional deficiencies and other CD-related health consequences avoidance, and mortality reduction. However, multiple factors influence GFD compliance, such as acceptance, access, availability, and cost of gluten-free products; dietary restrictions; socialization difficulties; and economic burden, among others, potentially negatively impacting CD QoL (5–9). In this sense, CD is considered a chronic condition that affects patients’ QoL as other chronic diseases (5–8) and, to achieve optimal health, it is essential to understand the individual’s perception of QoL (10). In chronic conditions, it is crucial to evaluate the impact of patients’ health conditions on their ability to live a fulfilling life and promote public policies to minimize the physical, emotional, and social burden on the patient (11, 12).

Studies have explored CD patients’ QoL perceptions using generic and specific questionnaires developed for celiac patients (13–22). The use of a specific questionnaire is important to comprehend aspects related to the celiacs’ QoL, mental health, well-being, and the economic and social aspects caused by this chronic condition and their lifelong dietary and lifestyle changes (11, 23). However, the use of a general questionnaire such as Short Form-36 (SF-36) may allow comparison among individuals with different chronic diseases or healthy individuals (24–27). The SF-36 is a widely recognized questionnaire designed to assess an individual’s health-related quality of life and functional abilities and is highly used as a generic instrument in gastroenterology (13, 28–30). Comprising 36 items that explore eight different aspects of QoL, it offers a detailed evaluation of physical functioning, limitations in daily activities due to physical health issues, pain levels, overall health perception, energy levels, social functioning, limitations in activities due to emotional problems, and mental health.

Considering the specific questionnaires to measure CD patients’ QoL, the Celiac Disease Questionnaire (CDQ) is broadly applied (11, 23) that used SF-36 in its validation process (13). CDQ was developed, validated and applied in Germany (2006) and later, it was translated and applied in several European and Extra-European countries (5, 6, 15, 23, 31–43). In Portugal, a study translated and validated the CDQ into Portuguese (41) and our previous study evaluated the quality of life (QoL) perception among Portuguese celiac patients using this Portuguese version of CDQ (42). Furthermore, a separate study conducted in Portugal utilized the general questionnaire SF-36 to assess the perception of QoL in a sample of 195 Portuguese celiacs regarding compliance with a gluten-free diet (GFD) (44). However, no study has compared a generic (SF-36) and a specific (CDQ) questionnaire to evaluate the perception of QoL among Portuguese celiac patients.

Therefore, this study aimed to compare the SF-36 v2 (generic) and CDQ (specific) questionnaires used to evaluate the QoL in celiac Portuguese adult individuals. The study is justified by the need to understand the differences between specific and generic questionnaires and how they could impact the evaluation of QoL in CD.

## 2 Materials and methods

### 2.1 Study design and instruments

This cross-sectional study used non-probabilistic sampling based on Portuguese celiac patients who accessed the online survey in 2022. The online data collection method was chosen due to the pandemic caused by SARS-CoV-2, making it impossible to use face-to-face interviews. In addition, it is considered a productive and cheap method to enroll participants and reach a more extensive sample (45, 46). The instrument was composed of 3 parts: (i) socioeconomic, health and GFD adherence questions; (ii) the SF-36 v2– Portuguese version (generic questionnaire) and (iii) CDQ (specific questionnaire) (5). The CDQ is a specific questionnaire to evaluate CD patients’ QoL. It was developed by Häuser et al. (5) and validated in Portugal by Lobão et al. (41). This questionnaire comprises 28 items divided into 4 domains (emotions, gastrointestinal symptoms, concerns, and social) evaluated by 7-point scale (from “1” - worst QoL perception to “7” - best QoL perception). The QoL general instrument used was the SF-36 v2 Portuguese version, validated in Portugal. It is an adaptation of the SF-36, which generates a physical component summary (PCS) and a mental one (MCS). This questionnaire has 36 items divided into 8 domains (1. Physical functioning, 2. Role limitations due to physical health, 3. Pain, 4. General Health, 5. Energy/fatigue, 6. Social functioning, 7. Role limitations due to emotional problems, 8. Emotional well-being) (47). It is a widely used generic, coherent, and easily administered QoL questionnaire.

We also collected sociodemographic characteristics (gender, age, marital status, educational level) and clinical variables (age at CD diagnosis, GFD compliance, use of antidepressants). The GDF compliance was self-reported since we do not have a validated instrument to evaluate GFD compliance in Portugal. Considering data collection occurred during the COVID-19 pandemic, it was not possible to validate a new instrument to evaluate it since the laboratory tests were limited. Therefore, we opt to use self-reported GFD compliance, as performed in other studies (34, 39, 48–52). Participants chose the option that best characterized their current diet regarding the question: “Do you follow a gluten-free diet?”. The response options were: 1) Never; 2) Rarely; 3) Sometimes; 4) Almost always (most of the time); 5) Always. Strict GDF compliance was considered for those who self-reported always adhering to a GFD whereas all others considered “gluten-exposed”. All the participants filled out both questionnaires.

### 2.2 Participants and ethics

The online instrument was inserted in the SurveyMonkey^®^ online platform. Individuals were invited to participate in the study by the Portuguese Celiac Association (Associação Portuguesa de Celíacos - APC) or via social media posting the link from February to May 2022. The inclusion criteria were as follows: a) Individuals aged >18 years diagnosed with celiac disease (CD) underwent a comprehensive diagnostic process, including clinical, serological, and histopathological assessments (specifically high upper digestive endoscopy with duodenal biopsies), along with genetic testing (HLA DQ2 and DQ8 analysis), in line with the ESsCD guideline (53). This criterion encompasses adults initially diagnosed with CD during childhood, adhering to the European Society for Pediatric Gastroenterology, Hepatology, and Nutrition (ESPGHAN) criteria (54)) and b) Participants were residents of Portugal and affiliated with the Portuguese Celiac Association (Associação Portuguesa de Celíacos - APC). After reading all the information about the study, those diagnosed with CD who agreed to participate accessed the questionnaire items. Those who disagreed were driven to the final page, acknowledging their time. All 234 individuals who signed the consent form to participate in the study completed the questionnaire.

The research followed the American Psychological Association (APA) Ethical Guidelines for Research involving Human Subjects. The participants were informed about the study’s scope, signed the informed consent form, and were not compensated for their participation. The Polytechnic University of Viseu Ethics Committee approved the ethical aspects of this study (n.° 59/SUB/2021 - 26th July 2021).

### 2.3 Statistical analysis

Data were extracted from the SurveyMonkey^®^ platform and evaluated using International Business Machines Corporation (IBM) Statistical Package for the Social Sciences (SPSS) Statistics, version 22 (Armonk, NY: IBM Corp). The statistical analysis considered the CDQ and SF-36 scores.

Descriptive statistics were performed as mean and standard deviation for SF-36 subscales. Student’s t-test, Analysis of Variance (ANOVA) and Tukey’s posthoc test were used to compare the SF-36 and the variables of interest. The tests considered two-tailed hypotheses and a significance level of 5%. The association between the CDQ and SF-36 V2 was verified using Spearman’s correlation.

## 3 Results

A total of 234 individuals accepted to participate in the study and completed the questionnaire. The questionnaire was virtually applied, and all individuals who accessed it completed it. **Table 1** shows data from the SF-36 domains subcategorized by sex, age, age at diagnosis, education, marital status, and diet. Males showed better scores for SF-36 domain 1 (Physical functioning), domain 2 (Role limitations due to physical health) and domain 7 (Role limitations due to emotional problems), and lower scores for domains 4 (General Health) and 5 (Energy/fatigue). Age differed only for domain 2 (Role limitations due to physical health), in which those > 40 y/o had better scores. Age at diagnosis differed only for domains 5 (Energy/fatigue), in which > 20 y/o at CD diagnosis had better scores and 6 (Social functioning) in which up to 20 y/o at CD diagnosis had better scores. Considering the educational level, participants with the highest educational level presented lower scores for domains 1, 2, 3, 6 and 7. Patients living alone presented lower scores for domain 1. Those following a GFD presented lower scores for D1, 2, 6, and 7 and the best score for D5 (Energy/fatigue). The use of antidepressants did not influence the SF-36 domains.

**Table 1:** SF-36 domains analyzed with subcategories based on sex, age groups, age at diagnosis of the condition, educational attainment, marital status, and dietary habits (n=234).

	D1 Physical functioning	D2 Role limitations due to physical health	D3 Pain	D4 General Health	D5 Energy/fatigue	D6 Social functioning	D7 Role limitations due to emotional problems	D8 Emotional well-being
	Mean (SD)	Mean (SD)	Mean (SD)	Mean (SD)	Mean (SD)	Mean (SD)	Mean (SD)	Mean (SD)
Gender*								
Female (n=162)	24.69 (23.22)^a^	34.72 (25.66)^a^	36.33 (24.13)^a^	51.93 (13.34)^b^	52.70 (19.32)^b^	38.35 (22.90)^a^	38.12 (25.36)^a^	46.94 (18.13)^a^
Male (n=66)	33.94 (24.50)^b^	46.97 (26.66)^b^	35.41 (23.04)^a^	47.47 (9.75)^a^	46.40 (11.13)^a^	43.37 (16.00)^a^	46.59 (24.76)^b^	46.36 (10.65)^a^
p	0.008	0.001	0.791	0.006	0.002	0.061	0.022	0.808
Age*								
Up to 40 y/o (n=132)	24.55 (23.89)^a^	37.36 (27.19)^a^	33.58 (22.67)^a^	50.02 (13.21)^a^	51.37 (18.26)^a^	39.77 (21.00)^a^	40.09 (26.81)^a^	47.73 (17.36)^a^
> 40 y/o (n=102)	32.45 (23.86)^b^	40.87 (25.52)^a^	39.39 (24.53)^a^	50.97 (11.58)^a^	49.88 (16.19)^a^	40.69 (21.45)^a^	42.40 (23.40)^a^	45.44 (14.28)^a^
p	0.013	0.315	0.062	0.567	0.515	0.744	0.490	0.282
Age at diagnosis*								
Up to 20 y/o (n=115)	30.65 (24.24)^a^	41.14 (26.58)^a^	35.89 (22.37)^a^	49.38 (10.85)^a^	47.72 (14.30)^a^	43.59 (19.05)^b^	44.49 (25.02)^a^	47.17 (13.77)^a^
> 20 y/o (n=116)	25.99 (23.90)^a^	37.66 (25.95)^a^	37.01 (24.78)^a^	50.93 (13.66)^a^	53.77 (19.64)^b^	37.39 (22.51)^a^	38.79 (24.92)^a^	46.77 (18.11)^a^
p	0.143	0.315	0.718	0.341	0.008	0.025	0.084	0.848
Educational level**								
Up to elementary school (n=35)	37.00 (22.00)^b^	50.18 (24.28)^b^	39.11 (20.09)^ab^	47.23 (11.40)^a^	48.04 (12.48)^a^	43.21 (17.24)^b^	50.24 (25.60)^b^	46.14 (11.89)^a^
High school (n=61)	41.15 (25.09)^b^	48.26 (26.10)^b^	39.97 (23.15)^b^	51.00 (10.30)^a^	50.20 (13.79)^a^	47.75 (18.61)^b^	46.86 (26.05)^ab^	50.41 (12.49)^a^
Undergraduate (n=89)	22.42 (22.02)^a^	34.06 (24.46)^a^	36.92 (25.53)^ab^	51.34 (14.28)^a^	52.04 (20.03)^a^	39.04 (23.14)^ab^	35.30 (22.30)^a^	45.45 (18.47)^a^
Post-graduation (n=49)	15.31 (17.63)^a^	27.93 (25.80)^a^	27.69 (21.42)^a^	50.39 (12.30)^a^	50.89 (19.31)^a^	30.61 (19.27)^a^	37.93 (26.85)^ab^	44.90 (17.69)^a^
p	0.000	0.000	0.035	0.411	0.707	0.000	0.004	0.219
Marital status*								
With partner (n=142)	30.53 (24.46)^b^	40.67 (25.71)^a^	37.56 (25.47)^a^	50.32 (12.90)^a^	50.92 (17.96)^a^	41.55 (21.81)^a^	42.02 (24.61)^a^	47.25 (16.42)^a^
(n=92)	24.08 (23.24)^a^	36.14 (27.53)^a^	33.88 (20.38)^a^	50.62 (11.94)^a^	50.41 (16.52)^a^	38.04 (20.04)^a^	39.67 (26.54)^a^	45.92 (15.64)^a^
p	0.046	0.202	0.246	0.857	0.825	0.216	0.491	0.538
Gluten-free diet*,***								
No (n=105)	37.67 (23.46)^b^	46.85 (24.58)^b^	38.95 (21.01)^a^	49.38 (10.23)^a^	47.32 (11.56)^a^	44.52 (17.84)^b^	46.75 (24.12)^b^	46.71 (12.38)^a^
Yes (n=129)	20.12 (21.79)^a^	32.41 (26.28)^a^	33.80 (25.40)^a^	51.29 (14.07)^a^	53.49 (20.58)^b^	36.63 (22.97)^a^	36.50 (25.50)^a^	46.74 (18.63)^a^
p	0.000	0.000	0.091	0.230	0.004	0.003	0.002	0.989
Antidepressants*								
No (n=218)	27.50 (23.92)^a^	37.99 (26.50)^a^	35.91 (23.68)^a^	50.50 (12.27)^a^	50.37 (17.01)^a^	39.56 (20.89)^a^	39.91 (25.06)^a^	46.33 (15.81)^a^
Yes (n=16)	34.69 (26.99)^a^	51.17 (23.63)^a^	38.88 (23.43)^a^	49.63 (15.87)^a^	55.47 (21.76)^a^	48.44 (23.66)^a^	57.29 (24.51)^b^	52.19 (19.41)^a^
p	0.251	0.054	0.629	0.789	0.258	0.105	0.008	0.161

* Student’s t-test.

**Anova with Tukey’s posthoc. Groups with the same letters do not differ significantly.

***Compliance with a gluten-free diet was considered participants’ responses of “always following the diet”.

The CDQ domains’ maximum scores can be 49 and 196 in total. **Table 2** shows that our sample presented the lowest score for social and gastrointestinal CDQ domains (23.03 ± 9.53 and 25.12 ± 8.81, respectively). Evaluating the associations, the SF-36 Domain 4 (general health) presented a negative association with all CDQ domains (**Table 2**). All the other domains showed positive associations with the CDQ.

**Table 2:** Mean and SD of SF-36 V2 scores and correlation between CDQ scale subscores.

		Correlation with CDQ subscales									
	Mean	Emotion 28.35 (7.60)		Social 23.03 (9.53)		Worries 26.77 (8.78)		Symptoms 25.12 (8.81)		Total 103.28 (31.15)	
	(SD)	Corr*	p	Corr*	p	Corr*	p	Corr*	p	Corr*	p
D1	27.99 (24.15)	0.380	0.000	0.534	0.000	0.342	0.000	0.404	0.000	0.473	0.000
D2	38.89 (26.47)	0.370	0.000	0.537	0.000	0.297	0.000	0.366	0.000	0.441	0.000
D3	36.11 (23.62)	0.246	0.000	0.319	0.000	0.207	0.001	0.239	0.000	0.291	0.000
D4	50.44 (12.50)	-0.300	0.000	-0.358	0.000	-0.267	0.000	-0.310	0.000	-0.357	0.000
D5	50.72 (17.37)	0.353	0.000	0.213	0.001	0.161	0.014	0.206	0.002	0.237	0.000
D6	40.17 (21.15)	0.482	0.000	0.565	0.000	0.330	0.000	0.359	0.000	0.500	0.000
D7	41.10 (25.35)	0.376	0.000	0.417	0.000	0.201	0.002	0.289	0.000	0.351	0.000
D8	46.73 (16.10)	0.460	0.000	0.348	0.000	0.291	0.000	0.185	0.005	0.357	0.000

*Spearman’s correlation coefficient.

D1. Physical functioning, D2. Role limitations due to physical health, D3. Pain, D4. General Health, D5. Energy/fatigue, D6. Social functioning, D7. Role limitations due to emotional problems, D8. Emotional well-being.

## 4 Discussion

This study recently evaluated the QoL perception of Portuguese celiac patients using a general questionnaire (SF-36) and compared its association with the specific questionnaire CDQ since CD symptoms and a lifelong GFD may significantly impact celiacs’ QoL. In our sample, about 45% of participants (n = 105) did not comply with the GFD, similar to data found in a previous study performed in Portugal in 2014 with 195 celiac patients, in which 47.7% did not comply with the GFD (44). The authors did not find an association between the QoL perception using the SF-36 and GFD compliance (44) and mentioned that it would be expected that GFD compliance would be positively associated with QoL. They list some potential explanations for their results: i) celiac patients who do not comply with the GFD were those who present milder symptoms, which do not significantly compromise their QoL; ii) those who did not comply with the GFD consider it less disruptive to their daily lives than that compliance with the GFD and iii) the possibility that the SF-36 was not sensitive enough to differentiate compliance with the GFD. In our study, celiacs not complying with the GFD showed the best scores for D1, D2, D6 and D7.

The D1(Physical functioning) scores were higher for males, > 40 y/o, those with the lowest educational levels, with partners and those not following the GFD. This SF-36 domain is important for identifying physical compromise in chronic diseases that impair common routine and exercise activities. A study (55) estimating the impact of chronic pain on patients’ QoL and found that the participants presented significantly lower mean QoL scores across all domains of the SF-36. The score for the D1 domain among the 78 chronic pain subjects was 31.8 ± 27.2 in comparison to scores of 94.0 ± 12.4 and 90.2 ± 18.9 from the general population in studies from England and Wales, respectively (p=0.001). Regarding CD, however, the impact of the condition on patients’ physical functioning has been poorly studied. Tiredness/fatigue are common manifestations described in CD (56), but they do not severely compromise physical abilities. Nonetheless, some celiac patients may experience neurological manifestations (neuropathy and ataxia), which might affect the physical domain to a certain extent. Peripheral neuropathy usually manifests as tingling, pain, and numbness, primarily in the hands and feet (57).

Two dimensions measure the impact of health limitations due to role limitations arising from physical health (D2) or emotional problems (D7), considering the type and amount of work performed, the necessity to reduce work, or the challenges faced in carrying it out. D2 scores were higher for males, those with the lowest educational levels, and those not following the GFD. The role limitations due to emotional problems (D7) presented the lowest scores for females, those complying with the GFD and not using antidepressants.

The Pain dimension (D3) measures the intensity and discomfort caused by pain and how this interferes with normal work. D3 dimension was only affected by educational level, in which those with the highest educational level showed the worst score. Abdominal pain is a frequent symptom in celiac individuals, although more frequently found in childhood (58). Although a strict GFD improves CD clinical manifestations such as abdominal pain (59), participants from our study presented low scores in D3, possibly related to the poor diet compliance found in our sample.

Energy/fatigue dimension (D5) showed higher scores for females, those complying with the GFD, and those with the age of diagnosis > 20 y/o. Although fatigue is often reported among celiac individuals, it usually improves once the GFD is implemented by the patient (56), which is in accordance with our finding that those compliant with the diet had better scores for this domain.

Social functioning (D6) was higher in those with age at diagnosis up to 20 years old, with the lowest educational levels, and who did not comply with the GFD. The finding that participants who did not comply with the GFD had higher scores for the social functioning domain is not surprising. As mentioned above, although the restriction of gluten from the diet is essential to good health in celiac patients, it interferes with social situations in the patients’ family, friends, and school/work environments (59). Wolf et al. (60) evaluated the association of QOL and GFD knowledge and adherence among 80 teenagers and adults. When asked about barriers to the GFD, 56% of adults and 70% of teens mentioned its adverse social impact. Feelings such as misunderstanding, embarrassment, stigma, exclusion, awkwardness, and guilt were expressed by participants (60).

The Emotional well-being dimension (D8) did not vary with sociodemographic data, or GFD compliance. Mental health problems have been documented in CD. Depression and/or depressive symptoms seem more frequent and/or severe in celiac patients than in healthy samples (61). Even though adherence to the GFD did not influence the D8 dimension in our sample, Sainsbury and Marques (61) suggest that poor diet adherence and self-reported depressive symptoms are associated, with the direction of causation being unclear. The authors mention that maintaining gluten on the diet may contribute to the appearance of a depressive state due to physiological mechanisms such as malabsorption of nutrients. On the other hand, being depressed compromises the individual’s ability to provide self-care and implement a safe GFD.

Males presented better scores than females on D1, D2 and D7, and worse on D4 and D5. These data differ from the previous study performed in Portugal (44) in which gender differed in D3, D5 and D8 with best results from males. Interestingly, the “general health” domain of SF-36 showed a negative association with all CDQ domains, contrary to what the authors of this study would have anticipated. A Turkish study (23) performed in 2015 with 81 celiac participants who answered the CDQ and SF-36 questionnaires showed a correlation between both questionnaires for all domains, similar to what Hauser (13) found in a study performed with 463 German celiac patients and Marchese (31) in a study performed in Italy with 171 celiac patients. An important factor to consider analyzing our results is that nearly half of the subjects in the sample (45%, n = 105) did not adhere to the GFD. It might be possible that the SF-36 does not accurately capture the influence of the GFD on the QoL of celiac patients, as previously demonstrated in a study conducted in Portugal (40). Consequently, this limitation could potentially impact the interpretation of results for questions related to the GFD. It is expected that the complete elimination of gluten from the diet leads to the remission of symptoms, normalization of intestinal histology and reduced risk for other health complications associated with CD (53), which are necessary for good health status.

Another interesting point to consider in this regard is question 2 from the SF-36 v2. “Compared to one year ago, how would you rate your health in general now?”. It might be reasonable to assume that this question, when applied to celiac individuals, would be influenced by diet compliance and time since the diagnosis. Patients who have received their diagnosis longer will probably have more tools to deal with difficulties related to the diet and the disease itself. There is evidence that more knowledge about CD and the diet, and support by health professionals and family improves the GFD compliance (53), all of which require time being diagnosed to be accomplished. Moreover, GFD effects on time until clinical improvement occurs and health depends on the length of time the patient remained undiagnosed due to the magnitude of intestinal mucosa damage (59).

This study presents some limitations. The sample comprised adult celiacs recruited using the snowball method by social media, leading to a possible selection bias due to a non-probabilistic sample. In this sense, our results may not represent the general Portuguese celiac people. In addition, despite the broad use of self-reported compliance to a GDF (34, 39, 48–52), we could not confirm the information (62), since data collection occurred online due to the COVID-19 pandemic restrictions, limiting the access to confirmation by laboratory tests. Despite the Portuguese Celiac Association has distributed the questionnaire to participants from all regions of Portugal to encompass the range of experiences and viewpoints of people living with CD in the country, the questionnaire did not ask for their exact location limiting the discussion about cultural and sociodemographic aspects.

## 5 Conclusions

In our study, seven out of the eight SF-36 v2 Portuguese Health Survey domains showed a positive correlation to the specific questionnaire CDQ. However, the “General Health” domain (domain 4) exhibited a negative correlation with the CDQ. Differences in content between the two instruments might be able to explain this finding, since the CDQ explores issues regarding specificities of CD and the lifelong GFD burden. Given that about half of the sample from this study displayed poor diet adherence, it is possible that the SF-36 could not reflect the impact of CD treatment - the complete elimination of gluten from the diet - on patients’ health. This is a possible explanation for this result. Nonetheless, this issue should be carefully evaluated in future research.

Specific validated questionnaires for CD individuals, such as the CDQ, contemplate social, economic, and clinical variables that permeate the patient’s life context. Therefore, these instruments may be more suitable for evaluating QOL in this public. However, using a general questionnaire such as the SF-36 would be indicated for comparing QOL between celiac patients and the general population or even between CD and other disease individuals. In this case, we recommend to parallelly assess GFD compliance for control.

## Data availability statement

The original contributions presented in the study are included in the article/supplementary material. Further inquiries can be directed to the corresponding authors.

## Ethics statement

The study was conducted in accordance with the American Psychological Association (APA) for research involving human subjects, and approved by the Ethics Committee of Polythecnic University of Viseu (59/SUB/2021). The studies were conducted in accordance with local and international laws and institutional guidelines. The participants provided their written informed consent to participate in this study.

## Author contributions

CC: Conceptualization, Data curation, Formal analysis, Funding acquisition, Investigation, Methodology, Project administration, Resources, Supervision, Validation, Writing – original draft, Writing – review & editing. RZ: Conceptualization, Methodology, Writing – original draft, Writing – review & editing. AR: Conceptualization, Formal analysis, Funding acquisition, Methodology, Writing – original draft, Writing – review & editing. EN: Formal analysis, Writing – review & editing. FR: Formal analysis, Funding acquisition, Investigation, Writing – review & editing. PF: Writing – original draft, Writing – review & editing. ET-L: Conceptualization, Data curation, Formal analysis, Funding acquisition, Investigation, Methodology, Project administration, Resources, Supervision, Validation, Writing – original draft, Writing – review & editing.
